# Longitudinal study evaluating the role of physical activity in modifying Parkinson's disease progression

**DOI:** 10.6026/973206300213612

**Published:** 2025-10-31

**Authors:** Deepak C Rathod, Samidha Utkarsh Kurdikar, Mounica Ratnala, Rajput Nixitsinh Arvindsinh, Priya Goyal

**Affiliations:** 1 Department of Clinical Haematology, University Hospital of Wales, Cardiff, United Kingdom; 2 Department of General Medicine, Healthway Hospital, Goa, India; 3 Department of Medicine, A.C.S.R Government Medical College, Nellore andhra Pradesh, India; 4 Department of Medicine, ITM hospital, Gwalior, Madhya Pradesh, India; 5 Department of Internal Medicine, Dayanand Medical College and Hospital, Ludhiana, Punjab, India

**Keywords:** Parkinson's disease, physical activity, disease progression, motor symptoms, UPDRS

## Abstract

The role of physical activity in modifying Parkinson's disease progression is of interest. This longitudinal study followed 118 patients with idiopathic Parkinson's disease over 24 months to evaluate the impact of physical activity on disease progression. Participants were grouped based on physical activity levels sedentary, moderately active and highly active using standardized activity questionnaires. Disease severity was assessed using the Unified Parkinson's Disease Rating Scale (UPDRS) and Hoehn and Yahr staging. Patients with higher physical activity levels showed slower motor and functional decline compared to sedentary individuals. Thus, we show that regular physical activity may positively influence the trajectory of Parkinson's disease.

## Background:

Parkinson's disease (PD) is a progressive neurodegenerative disease that is diagnosed clinically with the presence of bradykinesia, rigidity, tremor and postural instability [1]. Aside from the movement disorder aspects of PD, cognitive impairment, mood symptoms and autonomic dysfunction often develop in late stages of the disease, all contributing to reduced independence and quality of life [2]. While pharmacological therapy, specifically dopaminergic medications, is an important strategy in symptom management for PD, these therapies primarily manage symptoms and do not treat or reverse the neurodegenerative process [3]. Given this reality, additional, non-pharmaceutical treatment strategies should be considered for their potential to improve long term outcomes or, in some cases, influence the course and progression of the disease [4]. Exercise is one treatment option that has great potential as an adjunct intervention in the management of PD. Clinical research has shown that exercise training can improve movement function, balance, gait stability and overall health and wellbeing [5]. Observational studies of those living with PD also indicate that exercise was associated with better Quality of Life as compared to sedentary patients with PD [6]. At the mechanism level, preclinical studies have shown that exercise could have a neuroprotective role by promoting neuroplasticity, increasing neurotrophic factors, enhancing mitochondrial function and increasing striatal dopamine levels, while at the same time reducing neuroinflammation and oxidative stress [7]. Together, these biological mechanisms provide support to the hypothesis that physical activity may modify the neurodegeneration in PD rather than simply mitigate symptoms [8]. Notwithstanding this possibility, longitudinal studies assessing the influence of on-going physical activity on disease progression are limited and evidence is frequently inconsistent because of differences in the type, frequency, duration and intensity of exercise [9]. A clearer understanding of the long-term effects of physical activity on clinical outcomes, such as motor progression and disease staging, would provide important information on patient management and treatment recommendations [10]. Therefore, it is of interest to investigate the relationship between long-term levels of physical activity and progression of Parkinson's disease, specifically changes in clinical staging and motor function scores over a two-year follow-up period.

## Materials and Methods:

This longitudinal study was conducted at a tertiary neurology center over a 24-month period. A total of 118 patients diagnosed with idiopathic Parkinson's disease according to the UK Parkinson's Disease Society Brain Bank Criteria were enrolled. Inclusion criteria included age between 45 and 75 years, Hoehn and Yahr stage I-III at baseline and stable medication regimen for at least one month prior to study entry. Patients with atypical Parkinsonism, severe comorbidities, cognitive impairment (MMSE <24), or history of recent major surgery were excluded. Baseline data included demographic characteristics, disease duration, medication history and comorbidities. Physical activity levels were assessed using the Physical Activity Scale for the Elderly (PASE) and categorized as sedentary (PASE <70), moderately active (PASE 70-130) and highly active (PASE >130). Each patient underwent clinical evaluation using the Unified Parkinson's Disease Rating Scale (UPDRS) parts I-III and Hoehn and Yahr staging at baseline, 12 months and 24 months. Standardized exercise recommendations were provided to all participants; however, adherence was voluntary. Statistical analysis was performed using SPSS v26. Repeated-measures ANOVA were used to assess changes in UPDRS and Hoehn and Yahr scores over time across activity groups. Linear regression models were used to determine the independent effect of physical activity on disease progression, adjusting for age, sex, baseline disease severity and medication dosage. Ethical clearance was obtained and informed consent was collected from all participants.

## Results:

Of 118 individuals, 38 (32.2%) were deemed sedentary, 44 (37.3%) were moderately active and 36 (30.5%) were highly active. Throughout the 24-month follow-up, the highly active patients had statistically significantly decreased decline in clinician-rated motor status and functional insufficiency compared to sedentary patients. Sedentary participants had the steepest mean change in the UPDRS total sum, whereas the highly active patients had only minimally increased task-related mobility and cognitive engagement. Likewise, Hoehn and Yahr staging was associated with more rapid advancement in the sedentary than in the highly active group, further supporting a protective effect of physical activity. Similarly, motor sub-scores (e.g., bradykinetic scores) also had the steepest decline in the sedentary group and improved in the highly active group. Scores on MMSE measures of cognitive performance remained stable across the groups, of note, supporting a notion that the effects of physical activity may predominantly be benefitting motor functions, not cognition. Tremor scores increased in all groups yet less so in the highly active patients. Moreover, those engaged in structured aerobic exercise 3 times/week had the lowest mean rates of UPDRS progression and the lowest rate of functional loss. Sedentary patients also experienced significantly more falls, indicating even worse postural stability than either of the physically active groups. Multivariate regression models confirmed that physical activity was an independent predictor of slower progression of ICB after controlling for other factors. Finally, highly active patients required the smallest increases in levodopa dosage, demonstrating a tangible clinical benefit of sustained exercise in reducing treatment burden.

Table 1 (see PDF) shows the mean change in UPDRS total scores across physical activity groups over 24 months, demonstrating slower symptom progression among highly active participants. Table 2 (see PDF) presents the change in Hoehn and Yahr staging, highlighting that disease advancement was minimal in highly active individuals compared to sedentary patients. Table 3 (see PDF) shows the changes in UPDRS Part III motor scores, where sedentary patients had the greatest increases, indicating faster motor decline. Table 4 (see PDF) presents MMSE scores across groups, showing that cognitive decline was minimal and not significantly different among activity levels. Table 5 (see PDF) shows bradykinesia subscores, with highly active patients experiencing the slowest worsening compared to sedentary individuals. Table 6 (see PDF) presents' tremor score changes, indicating increases across all groups, but with less progression in the highly active group. Table 7 (see PDF) shows the impact of structured aerobic exercise frequency, where exercising three or more times weekly was linked to the least UPDRS deterioration. Table 8 (see PDF) presents the incidence of falls, revealing a higher frequency among sedentary patients compared to active participants. Table 9 (see PDF) shows the results of multivariate regression, confirming physical activity as an independent predictor of reduced UPDRS progression. Table 10 (see PDF) presents changes in levodopa equivalent daily dose, demonstrating that highly active patients required the smallest medication increase over 24 months.

## Discussion:

This longitudinal study provides compelling evidence that sustained physical activity is associated with a slower progression of Parkinson's disease (PD), particularly in motor function and overall disability. Participants who engaged in moderate to high levels of physical activity showed a significantly lower rate of UPDRS score increase and a more stable Hoehn and Yahr stage over two years, indicating that exercise may attenuate the clinical decline characteristic of PD [11]. These findings align with previous short-term trials and experimental data suggesting neuroprotective benefits of exercise through enhanced neuroplasticity, increased neurotrophic factors and improved dopaminergic transmission [12]. Importantly, the motor subcomponents most influenced by physical activity included bradykinesia and postural stability, both of which are major contributors to falls and loss of independence. The significantly lower incidence of falls in the active group reinforces the functional value of regular exercise in real-life settings [13]. Although tremor showed smaller changes between groups, the trend toward slower progression remained consistent. Cognitive function, as measured by MMSE, remained relatively stable in all groups, suggesting that while physical activity may preserve motor function, its effect on cognition might require longer observation periods or more sensitive measures [14]. Moreover, the observation that active individuals required smaller increases in dopaminergic medications further strengthens the clinical relevance of exercise as a non-pharmacological intervention capable of reducing medication burden and potentially minimizing side effects [15]. While this study adjusted for major confounders like age, baseline disease severity and medication dosage, self-reported physical activity and voluntary adherence may introduce some bias. Nevertheless, the findings support incorporating structured and personalized physical activity regimens into the standard care plan for PD patients, with potential long-term benefits for motor outcomes, medication needs and overall disease trajectory.

## Conclusion:

Regular physical activity is significantly associated with slower progression of motor symptoms in Parkinson's disease. Patients who maintained moderate to high levels of activity over two years showed reduced functional decline and required smaller increases in medication. Thus, we show integrating sustained physical activity as a core component of comprehensive Parkinson's disease management.
